# CXCL9 correlates with antitumor immunity and is predictive of a favorable prognosis in uterine corpus endometrial carcinoma

**DOI:** 10.3389/fonc.2023.1077780

**Published:** 2023-02-08

**Authors:** Shen Xue, Xiao-min Su, Li-na Ke, Yu-gang Huang

**Affiliations:** ^1^ Department of obstetrics and gynecology, Sinopharm Dongfeng General Hospital, Hubei University of Medicine, Shiyan, China; ^2^ Department of Immunology, Nankai University School of Medicine, Tianjin, China; ^3^ Department of Pathology, Taihe Hospital, Hubei University of Medicine, Shiyan, China

**Keywords:** uterine corpus endometrial carcinoma, CXCL9, anti-tumor immunity, PD-L1, immune microenvironment

## Abstract

**Background:**

The C-X-C motif chemokine ligand-9 (CXCL9) is related to the progression of multiple neoplasms. Yet, its biological functions in uterine corpus endometrioid carcinoma (UCEC) remain shrouded in confusion. Here, we assessed the prognostic significance and potential mechanism of CXCL9 in UCEC.

**Methods:**

Firstly, bioinformatics analysis of the public cancer database, including the Cancer Genome Atlas / the Genotype-Tissue Expression project (TCGA+ GTEx, n=552) and Gene Expression Omnibus (GEO): GSE63678 (n=7), were utilized for the CXCL9 expression-related analysis in UCEC. Then, the survival analysis of TCGA-UCEC was performed. Futher, the gene set enrichment analysis (GSEA) was carried out to reveal the potential molecular signaling pathway in UCEC associated with CXCL9 expression. Moreover, the immunohistochemistry (IHC) assay of our validation cohort (n=124) from human specimens were used to demonstrate the latent significance of CXCL9 in UCEC.

**Results:**

The bioinformatics analysis suggested that CXCL9 expression was significantly upregulated in UCEC patients; and hyper-expression of CXCL9 was related to prolonged survival. the GSEA enrichment analysis showed various immune response-related pathways, including T/NK cell, lymphocyte activation, cytokine-cytokine receptor interaction network, and chemokine signaling pathway, mediated by CXCL9. In addition, the cytotoxic molecules (IFNG, SLAMF7, JCHAIN, NKG7, GBP5, LYZ, GZMA, GZMB, and TNF3F9) and the immunosuppressive genes (including PD-L1) were positively related to the expression of CXCL9. Further, the IHC assay indicated that the CXCL9 protein expression was mainly located in intertumoral and significantly upregulated in the UCEC patients; UCEC with high intertumoral CXCL9 cell abundance harbored an improved prognosis; a higher ratio of anti-tumor immune cells (CD4^+^, CD8^+^, and CD56^+^ cell) and PD-L1 was found in UCEC with CXCL9 high expression.

**Conclusion:**

Overexpressed CXCL9 correlates with antitumor immunity and is predictive of a favorable prognosis in UCEC. It hinted that CXCL9 may serve as an independent prognostic biomarker or therapeutic target in UCEC patients, which augmented anti-tumor immune effects to furnish survival benefits.

## 1 Introduction

Uterine corpus endometrial carcinoma (UCEC) ranked as the third most popular gynecological malignant neoplasm worldwide. Merely in the United States, the estimated new cases and deaths of UCEC have reached 65,950 and 12,550, respectively, in 2022 (1). Approximately 75% of patients with UCEC are diagnosed at an early stage (International Federation of Gynecology and Obstetrics [FIGO] stages I or II), with an overall 5-year survival rate of approximately 80%, While UCEC patients diagnosed with advanced stage (stage III or IV) have an overall 5-year survival of about 60% or 25% (2). Currently, multiple therapeutic strategies, including surgery, chemotherapy, radiotherapy, and hormonal therapy, are still applied to UCEC therapy, but the incidence and mortality associated with the disease continue to increase annually (3, 4). Accurate assessment of prognosis is the cornerstone of effective treatment. However, patients with the same clinical stage may have different clinical features, suggesting that UCEC prognosis is not entirely accurate according to traditional clinicopathological staging (5). Immunotherapy has been an effective treatment for many cancers in recent years (6, 7), especially in non-small cell lung carcinoma (NSCLC) (8), melanoma (9), and colorectal cancer (10). The concept of UCEC as an immunogenic tumor has emerged (11), beginning with the observation that tumor-infiltrating lymphocyte subpopulations (TILs) are closely related to the prolongation of (OS) and progression-free survival (PFS) (12, 13). Therefore, the molecular mechanisms of the UCEC development should be further identified, and new biomarkers for prognostic assessment should be investigated. In addition to aberrant gene expression profiles, the tumor microenvironment (TME), particularly immune cells, is implicated in the occurrence and development of UCEC (14). Distinct roles for anti-tumor immunity depend on cytokine-cytokine interactions, which comprise the cytokine networks that commonly sustain homeostasis of the uterine corpus (15, 16). As a class of signaling cytokines, chemokines are prominent components in the interaction between tumor cells and the microenvironment, which interacts with receptors to regulate immune infiltration, tumor-associated angiogenesis, activation of the host immune response and tumor cell proliferation (17, 18). As a member of the chemokine superfamily that encodes secretory proteins implicated in the regulation of immune response and inflammatory process, the chemokine ligand C-X-C motif chemokine ligand-9 (CXCL9) is reported to be involved in T-cell trafficking, acting as a chemoattractant to lymphocytes but not neutrophils *via* the C-X-C motif 3 (CXCR3) pathway of the chemokine receptor (19). The CXCL9-CXCR3 signaling pathway involves in several physiological activities, including migration, differentiation, and activation of immune cells (20, 21). Overexpression of CXCL9 has been reported to associate with increased T-cell infiltration and prolonged OS in ovarian cancer (22). Based on the evidence of human samples and C57BL/6 mouse assay, hyper-expressed CXCL9 has been proven to stifle tumor cell growth and promote anti-PD-L1 therapy in ovarian cancer, especially the histological subtypes of clear-cell carcinomas (23). Chow et al. revealed that CXCL9 (derived from CD103^+^ dendritic cell) and CD8^+^ T cells (CXCR3^+^ are essential for the efficiency of anti-PD-1 therapy in melanoma, colon adenocarcinoma, and breast adenocarcinoma cell lines (21). However, the expression profile, masked prognosis significance, and molecular mechanism of CXCL9 in UCEC remain unclear. In addition, the CXCL9/10/11-CXCR3 signaling axis mainly regulates immune cell migration, differentiation, and activation. The immune response is triggered by the recruitment of immune cells, including cytotoxic lymphocytes (CTL), macrophages, natural killer cells (NK), and NK T cells. CXCL9 was crucial for the recruitment of NK and T cells, and facilitates interactions between DCs and T cells during immunotherapy (24). Tumor-infiltrating lymphocytes are the key to achieving a favorable prognosis and predicting response to existing checkpoint inhibitors. However, *in vivo* studies have demonstrated that the CXCL9-CXCR3 signaling axis also has tumorigenicity by enhancing tumor cell proliferation and metastasis (25). Therefore, more investigations on CXCL9 in the tumor microenvironment are required to learn whether it has any clinical relevance for immunotherapy or could serve as a predictive biomarker for UCEC.

## 2 Materials and methods

### 2.1 Patient UCEC specimens

In this study, three independent datasets were investigated, including the TCGA dataset (n=552), the GSE63678 dataset (n=7), and our validation cohort (n=124). All specimens in our validation cohort were gathered as formalin-fixed and paraffin-embedded (FFPE) specimens and diagnosed by two or three pathologists to evaluate pathologic characteristics. Finally, we collected 34 normal control endometrial tissues (corresponding normal tissues from leiomyoma or inflammation samples) and 90 UCEC samples from February 2019 to February 2022 for this study. All clinical samples and relevant information were gathered from patients or family members with informed consent (IFC). This study was supported and approved by the Ethics Committee of Taihe Hospital. Clinically relevant parameters of all selected patients were illustrated in **Supplementary Table S1**.

### 2.2 Immunohistochemistry (IHC) analysis

The IHC assay of all FFPE tissues was conducted according to the manufacturer’s protocol. Specifically, all 3 μm sections cut from FFPE were dewaxed with xylene and rehydrated with graded ethanol. Endogenous peroxidase activity was blocked with 3% hydrogen peroxide in methanol for 10 min. Sections were incubated with primary antibody (**Supplementary Table S2**) at 4°C overnight and with HRP-labeled secondary antibody for 0.5 h at 37°C, and hematoxylin staining was performed for 30 s at 37°C.

The protein expression was assessed *via* a combined score based on the intensity and the extent of staining under 200× field microscopy. Three experienced pathologists scanned and scored all IHC staining results, and the final score was taken as the median value. The assessing criteria for the extent of staining of positive cells were denoted as follows: 0 (absent), 1 (1%-10%), 2 (11%-50%), 3 (51%-80%) and 4 (81%-100%). The staining intensity scores were grad as 1 (weak), 2 (moderate), and 3 (strong). The quantity and intensity scores were multiplied to yield an overall score of 0 to 12. Finally, cases with scores of 0-3 were seated as a low-expression subgroup. And, cases with scores of 4-12 were recorded as a high-expression subgroup.

### 2.3 Quantitative real-time PCR

RT-qPCR analyzed the relative expression of the CXCL9 according to the merchant’s protocol. Total RNA was isolated from FFPE blocks by RNeasy FFPE Kit (QIAGEN, Germany). Total RNA of 1 μg was reversely transcribed into cDNAs using Revert-Aid First Strand cDNA Synthesis Kit (Thermo Scientific, USA). CXCL9 was amplified in a 20-μL volume that contained 2 μL of cDNAs, 1 μl of forward and reverse primers (10 nmol/L) and 10 μL of PowerUp SYBR Green Master Mix (Thermo Scientific, USA) for 38 cycles (95˚C for 15 s, 57˚C for 15 s, 72˚C for 30 s) after an initial 120 s denaturation at 95˚C in an ABI Prism 7500 analyzer (Applied Biosystems, USA). GAPDH was utilized as an endogenous reference gene. All reactions were run in triplicate. The relative *CXCL9* mRNA expression was assessed by using the 2^−ΔΔCt^ method. All primers were manufactured by Sangon Biotech (Shanghai, China), and the corresponding sequences were presented in **Supplementary Table S3**.

### 2.4 Immune cell infiltration signatures

Immune cell infiltration into tumors has emerged as a research hotspot as one of the crucial indicators for speculating on the effect of immunotherapy. The correlation between immune cell infiltration and CXCL9 mRNA expression was assessed by five algorithms, including ssGSEA, ESTIMATE, TIMER, TISIDB, and xCELL, based on TCGA-UCEC data. In addition, Spearman’s test was adopted to measure the correlation between CXCL9 expression and immune cell abundance.

### 2.5 Gene set enrichment analysis

For exploring the underlying enriched molecular pathways in CXCL9^high^ tumor samples, gene set enrichment analyses were investigated by the Molecular SignaturesDatabase of GSEA software (version 4.2.2; Broad Institute, USA). Then, three predefined gene sets, including ‘h.all.v7.2.symbols.gmt’, ‘c2.cp.go.v7.2.symbols.gmt’ and’c2.cp.kegg.v7.2.symbols.gmt’, was assessed to yield the enrichment score. The main statistical results of GSEA include two indicators (normalized enrichment score (NES) threshold: | NES |>1 and *P*-value< 0.05).

### 2.6 Differential expression analysis

The R package ‘limma’ was applied to analyze differentially expressed genes in UCEC with CXCL9^high^ versus CXCL9^low^ samples. The upregulated or down-regulated genes were defined with a fold change ≥1.5 and normalized *P*-value< 0.05.

### 2.7 Associations of CXCL9 expression with immunosuppressive-related genes

The associations of CXCL9 expression with several immunosuppressive-related genes, including CD274(PD-L1), CTLA4, HAVCR2, LAG3, PDCD1(PD-1), PDCD1LG2(PD-L2), TIGIT, and SIGLEC15 closely related to immunotherapy, were explored in UCEC. Further, the “ggstatsplot” package of R software was applied to analyze the correlation between CXCL9 expression and microsatellite instability (MSI) or tumor mutation burden (TMB) to predict the potential anti-tumor effect of immunosuppressants.

### 2.8 Statistical analysis

Statistical analyses were performed using GraphPad Prism 8.0 software (San Diego, CA) and SPSS 25.0 software (IBM SPSS Inc., Chicago, IL). Data were presented as the mean ± standard deviation (Mean ± SD). Comparisons between the 2 groups were performed by One-Way ANOVA or 2-tailed Student’s t-test. The Kruskal-Wallis test was used to assess the correlation of CXCL9 expression with clinical features. We deployed both uni-and multiple Cox regression assays to characterize the effect of CXCL9 expression on survival time and other clinical features. For prognostic analysis, the log-rank test was applied. The correlation between the two variables was tested using Pearson’s chi-square test.Statistical significance was established at a threshold of *P <*0.05 (ns=non-significant, * *P <*0.05, ** *P <*0.01, *** *P <*0.001).

## 3 Results

### 3.1 Aberrant expression of CXCL9 in UCEC, including various histomorphological subtypes and molecular subtypes

**Figure 1:**
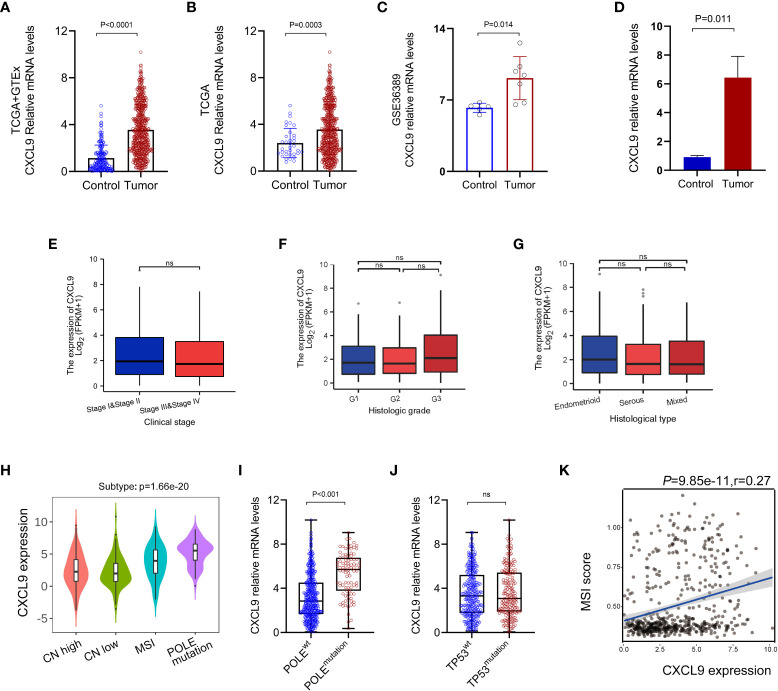
Aberrant expression pattern of CXCL9 in UCEC. The mRNA expression of CXCL9 in UCEC tumor and normal control tissues is based on the TCGA+GTEx dataset **(A)**, TCGA dataset **(B)**, GSE36389 **(C)**, and tissues of our validation cohort **(D)**. The mRNA expression of CXCL9 in different clinical stages **(E)**, histological grades **(F)**, and histological subtypes **(G)** based on TCGA-UCEC. The classical molecular subtypes of UCEC patients **(H)**, especially POLE mutation status **(I)** and TP53 mutation status **(J)** wt, wild-type; ns, non-significant. **(K)** The correlation analysis between the expression of CXCL9 and MSI score in UCEC patients.

The bioinformatics analysis was carried out to state that the CXCL9 expression was remarkably upregulated in UCEC tumor, compared with control ones based on the TCGA+GTEx dataset (*P*< 0.0001, **Figure 1A**), TCGA dataset (**Figure 1B**, *P*=0.0003), GSE36389 (**Figure 1C**, *P*=0.014) and tissues of our validation cohort (**Figure 1D**, *P*= 0.011). However, no difference was found in different clinical stages (**Figure 1E**, *P* > 0.05), histological grades (**Figure 1F**, *P*> 0.05), and histological subtypes (**Figure 1G**, *P* > 0.05) based on the TCGA-UCEC data repository. Further, the analysis of classical molecular subtypes was performed to reveal that the expression of CXCL9 was significantly different among CN-high (high-level somatic copy number alterations), CN-low, MSI, and POLE mutation subgroups in UCEC patients (**Figure 1H**, *P* < 0.01). Specifically, the CXCL9 was significantly overexpressed in POLE^mutation^ (POLE mutation-type), compared with POLE^wt^ (POLE wild-type) (**Figure 1I**, *P* < 0.001), but not between TP53^mutation^ and TP53^wt^ (**Figure 1J**, *P*> 0.05). Additionally, a tight correlation was depicted between the expression of CXCL9 and MSI score in UCEC (**Figure 1K**, *P*< 0.001, r=0.27).

### 3.2 Correlation between CXCL9 expression and clinicopathological signatures and prognostic value in UCEC patients

**Figure 2:**
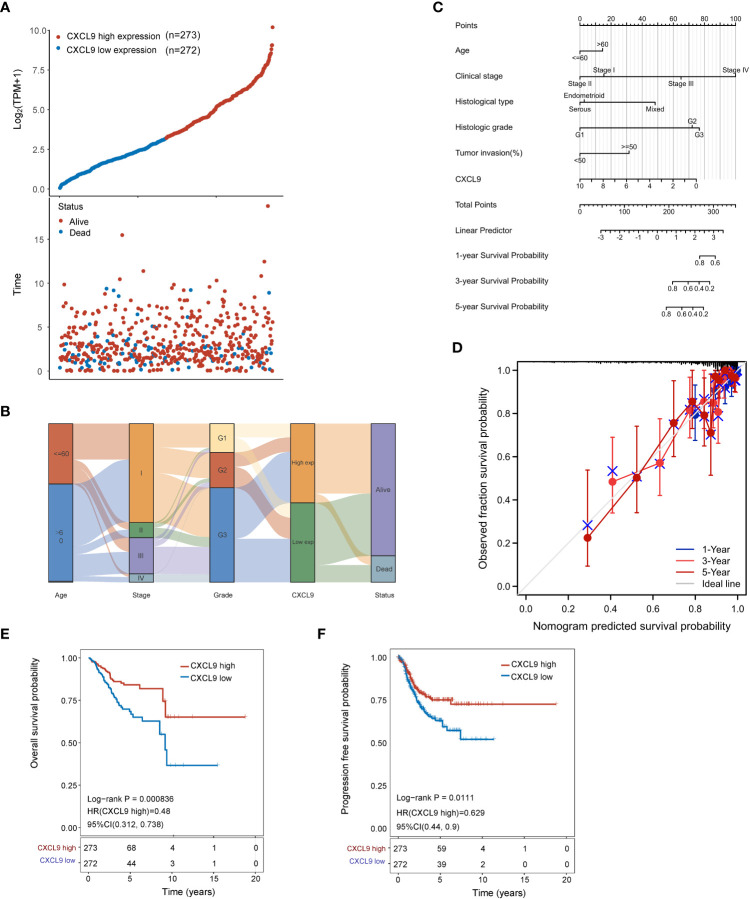
The prognostic assay of *CXCL9* expression in the clinicopathological parameters of UCEC patients. **(A)** *CXCL9* expression (low or high) and survival status (dead or alive) in UCEC patients. The order of related samples was consistent. **(B)** Sanguini plot for the analysis of the distribution of UCEC expression in age, stage, grade, and survival status. **(C)** Nomogram to predict 1-, 3- and 5-year survival rates in UCEC patients associated with *CXCL9* expression. **(D)** Calibration curves of the Nomogram model for the OS of UCEC patients. The diagonal dashed line denotes the ideal Nomogram, and the blue, orange, and red lines represent the 1-y, 3-y and 5-y observed Nomograms. The closer the Nomogram model matches the calibration curve, the better the model is at predicting the result. The survival assay of *CXCL9* expression in UCEC patients, including the OS **(E)** and PFS **(F)**.

All UCEC cases from TCGA were divided into two subgroups, including CXCL9^low^ (CXCL9 low expression, n=272) and CXCL9^high^ (CXCL9 high expression, n=273), according to the median cut-off to probe the correlation between *CXCL9* expression and clinicopathological signatures in UCEC (**Figure 2A**). As illustrated in **Table 1**, CXCL9 expression was independent of age, BMI, histological subtype, histologic grade, clinical stage, etc. (*P*>0.05). However, UCEC patients with CXCL9^high^ undergo a favorable outcome, compared with CXCL9^low^ subgroups in the TCGA dataset (*P*=0.017) and the validation cohort (**Supplementary Table S1**, *P*=0.008). Then, the Sanguini diagram depicted the distribution of *CXCL9* expression in age, TNM stages, grades, and survival status (**Figure 2B**). Further, the uni-cox and multi-cox regression assays were conducted to analyze the correlation between *CXCL9* and clinical factors, such as age and TNM stage, on the OS of UCEC. In the uni-cox analysis, *CXCL9* expression, age, clinical stage, histological subtype, histologic grade, tumor invasion, and residual tumor were closely related to OS in UCECC patients. In the multi-cox analysis, *CXCL9* expression, age, and the clinical stage could be independent prognostic factors for the OS of UCEC patients (**Table 2**, all *P*<0.05). Then, the Nomogram was performed to evaluate prognosis at 1, 2, or 3 years in UCEC patients with CXCL9^high^ (**Figures 2C, D**). Compared with the CXCL9^low^ subgroup, CXCL9^high^ indicates a better OS (**Figure 2E**; HR = 0.48, log-rank *P*=0.0000836) and PFS (**Figure 2F**; HR =0.629, log-rank *P*= 0.0111) based on the predictive analysis of the TCGA-UCEC dataset.

**Table 1:** Correlation between ESM1 expression and clinicopathological characteristics in UCEC patients (n=552).

Characteristic	Low expression of CXCL9	High expression of CXCL9	p value
n	276	276	
Age, n (%)			0.474
<=60	99 (18%)	107 (19.5%)	
>60	177 (32.2%)	166 (30.2%)	
BMI, n (%)			0.120
<=30	97 (18.7%)	115 (22.2%)	
>30	163 (31.4%)	144 (27.7%)	
Histological subtype, n (%)			0.094
Endometrioid	194 (35.1%)	216 (39.1%)	
Mixed	13 (2.4%)	11 (2%)	
Serous	69 (12.5%)	49 (8.9%)	
Histologic grade, n (%)			0.092
G1	51 (9.4%)	47 (8.7%)	
G2	69 (12.8%)	51 (9.4%)	
G3	149 (27.5%)	174 (32.2%)	
Clinical stage, n (%)			0.226
Stage I	161 (29.2%)	181 (32.8%)	
Stage II	31 (5.6%)	20 (3.6%)	
Stage III	70 (12.7%)	60 (10.9%)	
Stage IV	14 (2.5%)	15 (2.7%)	
Residual tumor, n (%)			0.870
R0	183 (44.3%)	192 (46.5%)	
R1	12 (2.9%)	10 (2.4%)	
R2	8 (1.9%)	8 (1.9%)	
Tumor invasion (%), n (%)			0.234
<50	137 (28.9%)	122 (25.7%)	
>=50	101 (21.3%)	114 (24.1%)	
Surgical approach, n (%)			1.000
Minimally Invasive	106 (20%)	102 (19.2%)	
open	163 (30.8%)	159 (30%)	
OS event, n (%)			0.017
Alive	218 (39.5%)	240 (43.5%)	
Dead	58 (10.5%)	36 (6.5%)	

**Table 2:** The univariate-cox and multivariate-cox regression analysis of CXCL9 and related clinical factors, including age, histologic grade and TNM stage, etc.

Characteristics	Total(n)	Univariate-cox analysis		Multivariate-cox analysis	
		Hazard ratio (95% CI)	P value	Hazard ratio (95% CI)	P value
Age	549				
<=60	206	Reference			
>60	343	1.847 (1.160-2.940)	0.010*	2.020 (1.036-3.939)	0.039*
Clinical stage	551				
Stage I& Stage II	392	Reference			
Stage III& Stage IV	159	3.543 (2.355-5.329)	<0.001*	5.475 (2.774-10.806)	<0.001*
Histological subtype	551				
Endometrioid	409	Reference			
Serous	118	2.667 (1.739-4.088)	<0.001*	0.583 (0.271-1.252)	0.166
Mixed	24	2.421 (1.036-5.655)	0.041*	1.820 (0.583-5.681)	0.303
Histologic grade	540				
G1&G2	218	Reference			
G3	322	3.281 (1.907-5.643)	<0.001*	1.725 (0.813-3.662)	0.155
Tumor invasion (%)	473				
<50	259	Reference			
>=50	214	2.813 (1.744-4.535)	<0.001*	1.357 (0.718-2.565)	0.347
Residual tumor	412				
R0	374	Reference			
R1	22	1.578 (0.630-3.955)	0.331	1.631 (0.619-4.293)	0.322
R2	16	5.527 (2.879-10.612)	<0.001*	1.963 (0.854-4.514)	0.112
CXCL9 expression	551				
Low	276	Reference			
High	275	0.546 (0.360-0.829)	0.004*	0.385 (0.206-0.718)	0.003*

*P < 0.05.

### 3.3 Tumor immune microenvironment in UCEC associated with CXCL9 expression

**Figure 3:**
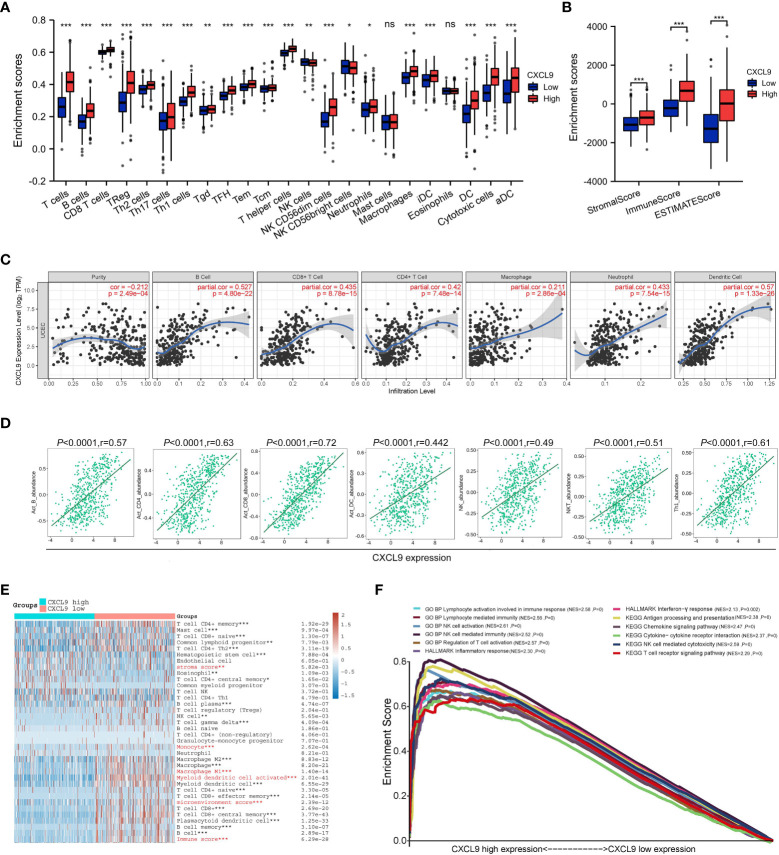
Immune cell infiltration analysis of *CXCL9* expression in UCEC patients with CXCL9^high^ and CXCL9^low^ subgroups. Assessment of relative abundance of tumor-infiltrating lymphocytes (TIL) in UCEC tissues with different CXCL9 mRNA expression status by ssGSEA **(A)**, ESTIMATE **(B)**, TIMER **(C)**, TISIDB **(D)**, and xCELL **(E)** based on TCGA dataset. **(F)** The GSEA enrichment analysis of UCEC patients with CXCL9^high^ versus CXCL9^low^ subgroups. ns=non-significant, * *P* <0.05, ** *P* <0.01, ***P < 0.001.

To probe the masked mechanism of CXCL9 in UCEC, we investigated the relationship between CXCL9 and tumor immune microenvironment based on the TCGA dataset. Five algorithms, including ssGSEA, ESTIMATE, TIMER, TISIDB, and xCELL, were performed to study immune cell infiltration associated with differential CXCL9 expression in UCEC. The ssGSEA figured out that overt differences of multiple immune cell populations were shown between the CXCL9^high^ and CXCL9^low^ subgroups of UCEC. The expression of CXCL9 was positively correlated with T cells, B cells, NK cells, DC cells, macrophages, etc., but not mast cells and eosinophils (**Figure 3A**). The ESTIMATE suggested that the enrichment of stromal score, immune score, and ESTIMATE score were dramatically different in the UCEC with CXCL9^high^ compared to the CXCL9^low^ (**Figure 3B**, all *P*<0.001). In **Figure 3C**, the TIMER demonstrated that CXCL9 expression level was negatively related to tumor purity (*P <*0.001, r=-0.212) but positively associated with B cells (*P*<0.001, r=0.527), CD8^+^ T cells (*P*<0.001, r=0.435), CD4^+^ T cells (*P*<0.001, r=0.42), macrophages (*P*<0.001, r=0.211), neutrophil (*P*<0.001, r=0.433), and DC (*P*<0.001, r=0.57). As shown in **Figure 3D**, the TISIDB asserted that CXCL9 expression was positively related to the abundance of Act B (activated B cells; r = 0.57, *P* < 0.001), Act CD4 (activated CD4 T cells; r = 0.63, *P*< 0.001), Act CD8 (activated CD8 T cells; r = 0.72, *P*< 0.001), Act DC (activated dendritic cells; r = 0.442, *P* < 0.001), NK (natural killer cells; r = 0.49, *P*< 0.001), NKT (natural killer T cells; r = 0.51, *P*< 0.001), and Th1 (activated dendritic cells; r = 0.61, *P*< 0.001) in UCEC. The xCELL depicted that CXCL9 high expression was mainly enriched in CD4^+^/CD8^+^ T cells, B cells, NK cells, monocyte, M1/M2-like macrophages, and DC, as well as different stroma score, immune score, and microenvironment score (**Figure 3E**). As presented in **Figure 3F**, the GSEA enrichment analysis of the GO: BP items showed that UCEC patients with CXCL9^high^ mainly were enrolled in the lymphocyte activation involved in immune response (NES=2.58, *P* =0), lymphocyte-mediated immunity (NES=2.56, *P* =0), NK cell activation (NES=2.61, *P*=0), NK cell-mediated immunity (NES=2.52, *P* =0), and regulation of T cell activation (NES=2.57, *P* =0). The GSEA analysis of the HALLMARK description uncovered that the overexpression of CXCL9 was tightly related to the inflammatory response (NES=2.30, *P*=0) and interferon-γ response (NES=2.13, *P*=0.002). The GSEA analysis of KEGG pathways pointed out that UCEC patients with CXCL9^high^ were significantly correlated with the antigen processing and presentation (NES=2.38, *P*=0), chemokine signaling pathway (NES=2.47, *P*=0), cytokine-cytokine receptor interaction (NES=2.37, *P*=0), NK cell-mediated cytotoxicity (NES=2.59, *P*=0), and T cell receptor signaling pathway (NES=2.29, *P*=0), which are widely known to augment the anti-tumor immunity.

**Figure 4:**
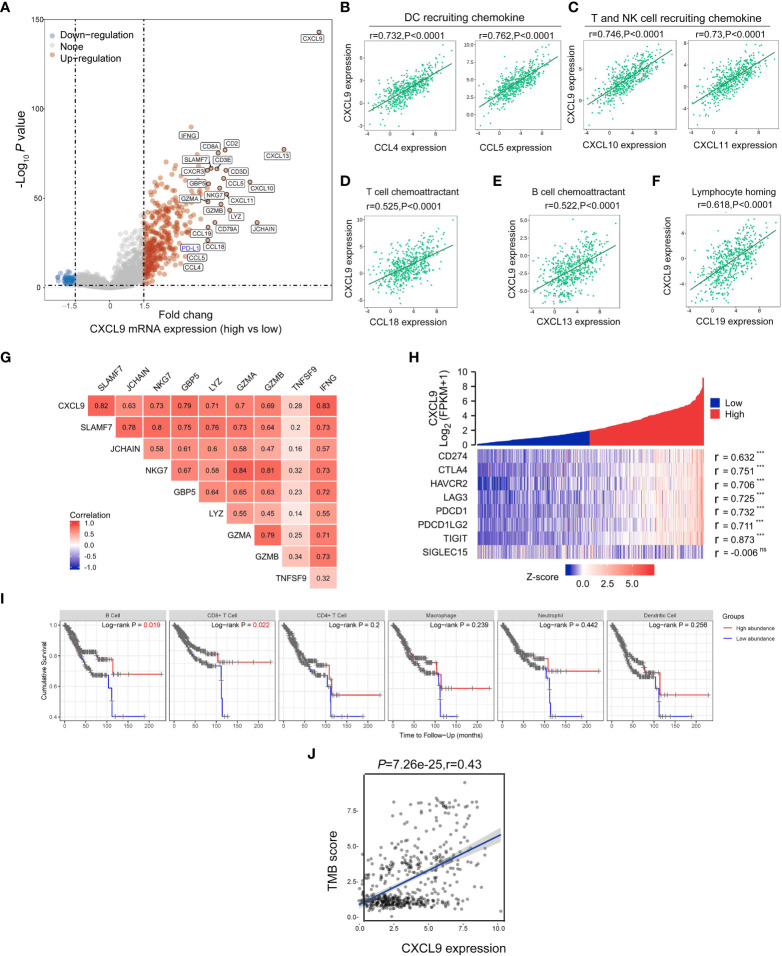
Correlations between *CXCL9* and immune-related genes in UCEC. **(A)** The differentially expressed genes in UCEC patients with CXCL9^high^ versus CXCL9^low^ subgroups visualized by a volcano plot. **(B–F)** Relationship of *CXCL9* expression to the chemokine gene-set related to DC recruiting, T cell and NK cell recruiting, T cell chemoattractant, B cell chemoattractant, and lymphocyte homing. Relationship between CXCL9 and cytotoxic molecules **(G)**, or immunosuppressive-related genes **(H)**. **(I)** The OS analysis of immune cell abundance in UCEC based on the TIMER service platform. **(J)** Correlations between *CXCL9* expression and TMB score in UCEC patients. ns=non-significant, ***P < 0.001.

Then, the differentially expressed genes in UCEC patients with CXCL9^high^ versus CXCL9^low^ subgroups were studied to find that high CXCL9 mRNA expression was remarkably related to various immune-activated genes. As presented in **Figure 4A** *via* the volcano plot, the CXCL9^high^ was enriched in the expression of IFNG, CXCL13, CXCL11, CXCL10, CCL4, CCL5, CCL18, CCL19, CD8A, and PD-L1. As illustrated in **Figures 4B–F**, CXCL11 was positively associated with the chemokine gene-set related to DC recruiting (CCL4: r=0.732, *P*<0.0001; CCL5: r=0.763, *P*<0.0001), T cell and NK cell recruiting (CXCL10: r=0.746, p<0.0001; CXCL11: r=0.73, *P*<0.0001), T cell chemoattractant (CCL18: r=0.525, *P*<0.0001), B cell chemoattractant (CXCL13: r=0.522, *P*<0.0001), and lymphocyte homing (CCL19: r=0.618, *P*<0.0001). To some extent, CXCL9 was identified to facilitate the antitumor immune microenvironment in UCEC. Additionally, the cytotoxicity molecules, including SLAMF7, JCHAIN, NKG7, GBP5, LYZ, GZMA, GZMB, TNF3F9, and IFNG, were positively related to the expression of CXCL9 mRNA expression (**Figure 4G**; Spearman’s r = 0.82, 0.63, 0.73, 0.79, 0.71, 0.7, 0.69, 0.28, and 0.83, respectively; all *P*< 0.01). Furthermore, several immunosuppressive-related genes, including CD274(PD-L1), CTLA4, HAVCR2, LAG3, PDCD1, PDCD1LG2, and TIGIT, were positively associated with CXCL9 expression (**Figure 4H**; Spearman’s r = 0.632, 0.751, 0.706, 0.725, 0.732, 0.711, and 0.873, respectively; all *P*< 0.001), but not SIGLEC15. Then, the OS analysis of TIMER suggested that high immune cell abundance, especially B cell (*P*=0.019) and CD8^+^ T cell (*P*=0.022), indicates a beneficial clinical outcome (**Figure 4I**). Lastly, it implied that CXCL9 expression was positively correlated with the TMB score in UCEC (**Figure 4J**; r=0.43, *P*<0.0001).

### 3.4 Aberrant expression and prognosis of CXCL9 in our validation cohort

**Figure 5:**
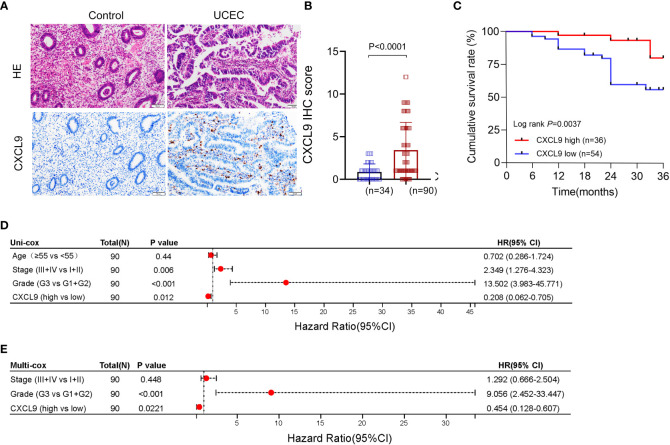
The expression and prognosis of CXCL9 in UCEC patients from the validation cohort. **(A, B)** The different expression of CXCL9^+^ cells in tumor and control samples. **(C)** The cumulative survival analysis of UCEC patients with CXCL9^high^ versus CXCL9^low^ subgroups. **(D, E)** The uni-cox and multi-cox regression analyses of CXCL9^+^ cell infiltration and clinicopathological variables in UCEC patients.

To further investigate the role of CXCL9, the UCEC samples cohort of our hospital was collected. Firstly, the protein expression of CXCL9 in tumor tissue was upregulated, compared with control tissue (**Figures 5A, B**) by IHC assay. Then, all patients were divided into two subgroups, high-expression and low-expression, based on the overall IHC score, and the respective clinical prognosis was analyzed. It suggested that UCEC patients with high CXCL9 abundance in tumor interstitial tissue featured a significantly better cumulative survival within the validation cohort (**Figure 5C**, *P*=0.0023). Furthermore, based on the uni-cox and multi-cox regression analyses, interstitial CXCL9 expression was strongly associated with UCEC patients’ survival and could be used as an independent prognostic biomarker for UCEC patients (**Figure 5D**; HR = 0.208; 95% CI 0.062–0.705; *P*= 0.012. **Figure 5E**, HR = 0.454; 95% CI 0.128–0.607; *P*= 0.0221).

### 3.5 Validation of CXCL9^+^ cells for association with immune cells and PD-L1

**Figure 6:**
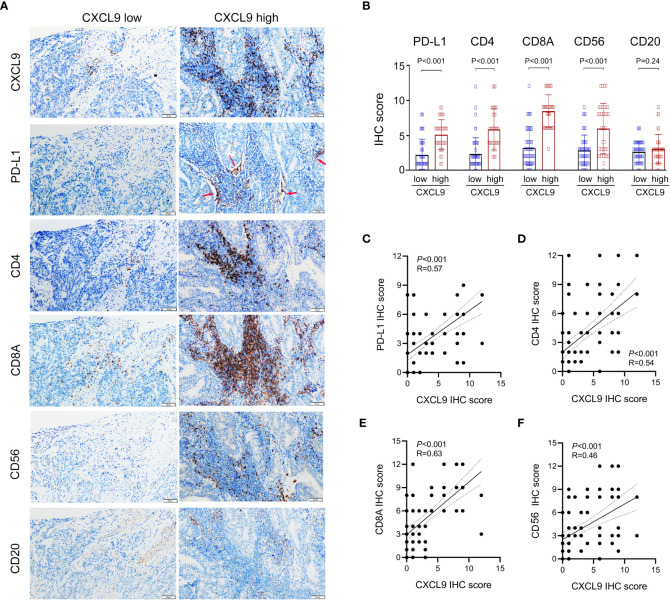
Identifying the correlations between the expression of CXCL9 and the abundance of PD-L1 and tumor-infiltrating lymphocytes (CD8^+^ T cell, CD4^+^ T cell, CD56^+^ NK cell, and CD20^+^ B cell) in UCEC patients *via* immunohistochemistry. **(A, B)** Comparison of the infiltration of PD-L1^+^, CD8A^+^, CD4^+^, CD56^+^, and CD20^+^ cells between CXCL9^high^ versus CXCL9^low^ subgroups in the validation cohort. **(C–F)** Correlation of CXCL9 with PD-L1, CD4, CD8A, and CD56 in UCEC patients.

Furthermore, the immunomodulatory function of CXCL9^+^ cell infiltration was assessed in the validation cohort. As presented in **Figure 6A**, tumors with significant infiltration of CXCL9+ cells showed significant intertumoral CD8^+^ T cells, CD4^+^ T cells, and CD56^+^ NK cells, which were related to antitumor immunity but not CD20^+^ B cells. Additionally, high levels of CXCL9^+^ cell infiltration were associated with the high level of PD-L1^+^ cells, which were expressed primarily at the junction between tumor cells and stroma. Thus, the expression of PD-L1, CD8A, CD4, and CD56, but not CD20, was higher in the CXCL9^high^ group than in the CXCL9^low^ group (**Figure 6B**). Within the validation cohort, it suggested that CXCL9 expression was positively associated with PD-L1, CD4, CD8A and CD56 (**Figures 6C–F**; r = 0.57, *P*< 0.001; r = 0.54, *P*< 0.001; r = 0.63, *P*< 0.001; r = 0.46, *P*< 0.001, respectively).

## 4 Discussion

UCEC is the most common gynecological neoplasm in high-income countries, and its incidence is rising worldwide (26). Based on clinical and endocrine characteristics (e.g., types I and II), histopathological signatures (e.g., endometrioid, clear-cell, or serous adenocarcinoma), or molecular subtypes (e.g., microsatellite instability, TP53 mutation, POLE mutation, or high-level somatic copy number alterations, HER2 amplification), it reveals the histological heterogeneity and complexity of this neoplasm from different perspectives (27, 28). Additionally, the homeostasis between tumor cells and tissue microenvironment (especially immune cells) played a crucial role in the tumorigenesis and progression of tumors (14). Cytokines are soluble proteins that arrange cell migration according to a defined concentration gradient. Cytokines form the immune landscape of the tumor microenvironment during the early stages of tumor development. CXCL9, also known as INF gamma (MIG) -induced monocytes, can be produced in inflammatory conditions by antigen-presenting cells (e.g., dendritic cells or macrophages) or tumor cells in the tumor microenvironment (29). It attracts cells expressing CXCR3 receptors, including activated T and NK cells, and has been proven closely related to the response to immune checkpoint therapy. Overexpressed CXCL9 has also been reported to impede tumor progression and metastasis by inhibiting angiogenesis (30). The unique roles of antitumor immunity are closely related to the cytokine-cytokine interactions, which form the cytokine networks to maintain the homeostasis of the uterine corpus (15, 16).

In this study, we found that the CXCL9 expression was dramatically upregulated in UCEC tumors, compared with control ones based on the TCGA dataset, GSE36389, and human tissues of our validation cohort. The overexpression of CXCL9 has independent prognostic value for UCEC. Furthermore, the results showed that upregulation of CXCL9 expression acts as a defense against the tumorigenesis or progression of UCEC.

In different cancers, CXCL9 serves as a tumor suppressor or promoter, as it may regulate cellular processes in a specific manner through different regulatory networks. The expression of CXCL9 could be induced by IL-27, IFN-γ, D-galactosamine, etc., *via* JAK/STAT1, NF-κB, Fra-1, and Eg-1 signaling pathways. Moreover, CXCL9, as one of the momentous chemoattractants for leukocytes, B cells, and T cells, can serve as a tumor suppressor (31). Zhang et al. reported that a combination of CXCL9 gene therapy with low-dose cisplatin improved therapeutic efficacy in colon carcinoma (CT26) and Lewis lung carcinoma (LL/2c) murine models *via* inhibiting angiogenesis, augmenting CTL infiltration, and showed thymus-dependent antitumor effects (32). Hoch et al. reported that the tumor microenvironment (TME) enriched by CXCL9 and CXCL10 contributes to the generation of a “hot” tumor microenvironment and predicts favorable OS in melanoma (33). Marcovecchio et al. showed that CXCL9-expressing tumor-associated macrophages enhance anti-PD(L)-1 response rates by regulating the recruitment of stem-like CD8 T cells, thus furnishing a novel clue to fight against cancer (34). Furthermore, Tokunaga et al. demonstrated that the CXCL9, -10, -11/CXCR3 axis regulates immune cell migration, differentiation, and activation through the paracrine axis (25). Thus, determining the type of this signaling axis is a potential target for cancer therapy in preclinical studies. Conversely, previous studies also identified that CXCL9 might act directly on multiple types of tumor cells expressing the CXCR3 receptor to improve epithelial-mesenchymal transition (EMT) and cell migration. Ding et al. suggested that CXCL9 boosted the migration and invasion of CD133^+^ liver cancer cells by activating the p-ERK1/2-MMP2/MMP9 pathway (35). Mir et al. demonstrated that elevated serum levels of CXCL9 indicated a shorter median event-free survival in follicular lymphoma patients (36). Amatschek et al. revealed that a low concentration of CXCL9 induced melanoma cell migration, while conversely at a high concentration (37).

Tumor-infiltrating immune cells are closely associated with tumorigenesis, tumor cell growth, and metastasis, which in turn regulate immune cell population and differentiation (38, 39). Evidence indicates that tumor progression may result from the escape of cancer cells from host immunosurveillance (40). Thus, clarifying the infiltrating immune cells in the TME may be able to enunciate the masking mechanism involving CXCL9 in UCEC. Based on the bioinformatics strategy, it showed that a higher ratio of anti-tumor immune cells characterized CXCL9 high expression. Further, the GSEA enrichment analysis showed multiple immune response-related pathways, including T/NK cell, lymphocyte activation, cytokine-cytokine receptor interaction network, and chemokine signaling pathway, mediated by CXCL9 high expression. Further, CXCL9 was positively related to CCL4, CCL5, CXCL10, and CXCL11 in UCEC, which are associated with DC, NK, and T cell recruitment and play an essential role in suppressing tumor growth and improving prognosis (41–43). Meanwhile, CXCL9 was also positively correlated with CXCL13, CCL18, and CCL19, which were closely related to the chemoattractant of B cell (44), T cell (45), and lymphocyte homing (46, 47). Then, positive correlations were found between CXCL9 and several cytotoxic molecules (SLAMF7, JCHAIN, NKG7, GBP5, LYZ, GZMA, GZMB, TNF3F9, and IFNG) that were shown by other immunocytes to promote cytotoxic function and contribute to immune-promoting (48). These results suggest that CXCL9 promotes anti-tumor immunity by mediating immune cell infiltration. Moreover, it suggested that CXCL9 expression positively correlated with PD-L1 in the TCGA and our validation cohort. CXCL9 expression was positively correlated with the TMB score in UCEC (**Figure 4J**). These discoveries were consistent to some extent with the finding that inhibition of CXCL9 expression or protein activity will weaken the therapeutic effect of anti-PD-1/PD-L1 therapy and lead to a dismal prognosis of melanoma patients (19). Since other immunosuppressants, including PD-1(PDCD1), CTLA4, HAVCR2, LAG3, PD-L2, and TIGIT, were positively correlated with CXCL9 mRNA expression, the related mechanism between CXCL9 and immune environment requires further study. Several pieces of literature have suggested that infiltration of large numbers of immune cells (e.g., CD8^+^T cells, NK cells, and DC cells) into tumor stroma can enhance the anti-tumor effect of immune checkpoint inhibitors (ICIs) (41, 49, 50). Nevertheless, anti-PD-1 therapy could not reduce the tumor growth in breast cancer and melanoma mouse models with CXCR3 knock-out treatment (51). Consequently, it is meaningful and promising to verify whether CXCL9 can infer the response of UCEC patients to ICIs. However, there are still some limitations of this paper. First, the specific cell population of CXCL9^+^ was not sorted out. Furthermore, the specific molecular mechanisms and exact functions of CXCL9 anti-tumor effects in UCEC need to be further explored and validated by *in vivo* and *in vitro* experiments.

## 5 Conclusion

In this study, we verified that overexpressed CXCL9 as an independent prognostic biomarker in UCEC patients augmented anti-tumor immunity and was related to significantly prolonging survival. Additionally, hyper-expression of PD-L1 associated with high-expression CXCL9 in UCEC may enhance the treatment response of patients to ICIs. Therefore, the above results can furnish new insights for clinicians to choose suitable treatment strategies for patients and improve the long-term outcomes of UCEC patients.

## Data availability statement

The original contributions presented in the study are included in the article/**Supplementary Material**. Further inquiries can be directed to the corresponding authors. All publicly available datasets involved in this work are from TCGA (https://portal.gdc.cancer.gov/) and GEO (https://www.ncbi.nlm.nih.gov/geo/).

## Ethics statement

The studies involving human participants were reviewed and approved by the Ethics Committee of Taihe Hospital. The patients/participants provided their written informed consent to participate in this study.

## Author contributions

SX and Y-GH conceived and designed the study and wrote the paper. Y-GH and L-NK supervised the research. X-MS offered helpful advice on methods and chart preparation. All authors contributed to the article and approved the submitted version.
