# Supplementary material for: CXCL9 correlates with antitumor immunity and is predictive of a favorable prognosis in uterine corpus endometrial carcinoma
Source: Front Oncol. 2023 Feb 8;13:1077780. doi: 10.3389/fonc.2023.1077780 (PMC9945585; doi:10.3389/fonc.2023.1077780)
Supplement: Supplementary file 1 [file Table_1.docx]

**Table S1. Correlation between CXCL9 expression and clinicopathological characteristics in UCEC patients (n=90).**

| **Characteristic** | **CXCL9 IHC score** | | **p value** | **Characteristic** | | **CXCL9 IHC score** | | | **p value** |
| --- | --- | --- | --- | --- | --- | --- | --- | --- | --- |
|  | High (n=36) | Low (n=54) |  |  |  | High (n=36) | | Low (n=54) |  |
| **Age** |  |  | 0.010 | **Clinical grade** | |  | |  | < 0.001 |
| Median (IQR) | 56.5 (52, 63) | 62 (56, 66) |  | Grade 1 | | 13 (14.4%) | | 3 (3.3%) |  |
| **Status** |  |  | 0.008 | Grade 2 | 16 (17.8%) | | 23 (25.6%) | |  |
| Alive | 33 (36.7%) | 35 (38.9%) |  | Grade 3 | | 7 (7.8%) | | 28 (31.1%) |  |
| Dead | 3 (3.3%) | 19 (21.1%) |  |  | |  | |  |  |
| **Pathologic stage** |  |  | 0.001 |  | |  | |  |  |
| Stage I+ II | 31（34.5%） | 15（16.6%） |  |  | |  | |  |  |
| Stage II+IV | 5（5.5%） | 39(43.3%) |  |  | |  | |  |  |
