# Supplementary material for: CXCL9 correlates with antitumor immunity and is predictive of a favorable prognosis in uterine corpus endometrial carcinoma
Source: Front Oncol. 2023 Feb 8;13:1077780. doi: 10.3389/fonc.2023.1077780 (PMC9945585; doi:10.3389/fonc.2023.1077780)
Supplement: Supplementary file 2 [file Table_2.docx]

**Table S2. Details of all antibodies involved in this study.**

| **Antibodies** | **Manufacturer brand** | **Catalogs NO.** | **Dilute proportion** |
| --- | --- | --- | --- |
| CXCL9 antibody | Abcam, UK | ab290643 | IHC,1:300 |
| PD-L1(22C3) | Agilent Dako, Denmark | SK006 | IHC, ready to use reagent |
| CD4 | Maxim biotechnologies, China | RMA-0620 | IHC, ready to use reagent |
| CD8A | Maxim biotechnologies, China | MAB-0021 | IHC, ready to use reagent |
| CD20 | Maxim biotechnologies, China | Kit-0001 | IHC, ready to use reagent |
| CD56 | Maxim biotechnologies, China | MAB-0743 | IHC, ready to use reagent |
