# Supplementary material for: CXCL9 correlates with antitumor immunity and is predictive of a favorable prognosis in uterine corpus endometrial carcinoma
Source: Front Oncol. 2023 Feb 8;13:1077780. doi: 10.3389/fonc.2023.1077780 (PMC9945585; doi:10.3389/fonc.2023.1077780)
Supplement: Supplementary file 3 [file Table_3.docx]

**Table S3.** The sequence information of RT-qPCR primers.

| **Gene name** | **Primers (5**'-**3**') | |
| --- | --- | --- |
| CXCL9 | Forward: CCAGTAGTGAGAAAGGGTCGC | Reverse: AGGGCTTGGGGCAAATTGTT |
| GAPDH | Forward: CGGAGTCAACGGATTTGGTCGTAT | Reverse: AGCCTTCTCCATGGTGGTGAAGAC |
